# Significance of Serum Oxidative and Antioxidative Status in Congenital Central Hypoventilation Syndrome (CCHS) Patients

**DOI:** 10.3390/antiox11081497

**Published:** 2022-07-30

**Authors:** Elisabetta Bigagli, Maura Lodovici, Marzia Vasarri, Marta Peruzzi, Niccolò Nassi, Donatella Degl’Innocenti

**Affiliations:** 1 Department of Neuroscience, Psychology, Drug Research and Child Health (NEUROFARBA), Section of Pharmacology and Toxicology, University of Florence, Viale Pieraccini 6, 50139 Florence, Italy; elisabetta.bigagli@unifi.it (E.B.); maura.lodovici@unifi.it (M.L.); 2 Department of Experimental and Clinical Biomedical Sciences “Mario Serio”, University of Florence, Viale Morgagni 50, 50134 Florence, Italy; marzia.vasarri@unifi.it; 3 Sleep Breathing Disorders and SIDS Centre, A. Meyer Children’s Hospital, Viale Pieraccini 24, 50139 Florence, Italy; marta.peruzzi@meyer.it (M.P.); niccolo.nassi@meyer.it (N.N.)

**Keywords:** CCHS, rare disease, oxidative stress, circulating biomarkers, TBARS, FRAP, AOPP

## Abstract

Congenital central hypoventilation syndrome (CCHS) is a rare neurological genetic disorder that affects sleep-related respiratory control. Currently, no drug therapy is available. In light of this, there is a need for lifelong ventilation support, at least during sleep, for these patients. The pathogenesis of several chronic diseases is influenced by oxidative stress. Thus, determining oxidative stress in CCHS may indicate further disorders in the course of this rare genetic disease. Liquid biopsies are widely used to assess circulating biomarkers of oxidative stress. In this study, ferric reducing ability of plasma, thiobarbituric acid-reactive substances, advanced oxidation protein products (AOPPs), and advanced glycation end-products were measured in the serum of CCHS patients to investigate the relationship between oxidative stress and CCHS and the significance of this balance in CCHS. Here, AOPPs were found to be the most relevant serum biomarker to monitor oxidative stress in CCHS patients. According to this communication, CCHS patients may suffer from other chronic pathophysiological processes because of the persistent levels of AOPPs.

## 1. Introduction

The development and the progression of several chronic diseases are often associated with oxidative stress (OS), a phenomenon defined as an imbalance between the formation of reactive oxygen species (ROS) and antioxidant defense systems [1,2]. ROS are biomarkers of OS; however, being highly reactive and with a short half-life, the measurement of the products of oxidative damage caused by ROS to lipids, proteins, and nucleic acids is the preferred approach to assess oxidative stress in clinical samples [3].

Advanced oxidation protein products (AOPPs), derived from the action of hypochlorous acid and chloramine produced by neutrophil myeloperoxidase, are regarded as both oxidative stress and inflammatory biomarkers in clinical samples [4]. Lipid peroxidation is commonly used as an indicator of ROS-induced damage to polyunsaturated fatty acids in the cell membrane [5]. Malondialdehyde (MDA) is one of the best-studied end-products of lipid peroxidation and can be measured using thiobarbituric acid-reactive substances (TBARS) [6].

Advanced glycation end-products (AGEs) are heterogeneous compounds generated from the nonenzymatic glycation of macromolecules (proteins, nucleic acids, and lipids) through the Maillard reaction; despite being often associated with hyperglycemia, increased AGEs have also been documented in normoglycemic subjects suffering from other conditions characterized by increased OS such as inflammatory diseases and obstructive sleep apnea [4,7].

Physiological homeostasis is maintained by sophisticated enzymatic and nonenzymatic antioxidant defenses that counteract excessive ROS levels [8]. The FRAP assay measures the ferric reducing ability of plasma, provides estimates of the total nonenzymatic antioxidants in plasma, and represents an indication of the intrinsic ability of plasma to prevent and limit the negative effects of oxidative stress [9]. FRAP values have been found to correlate with the clinical status in several metabolic, cardiovascular, and respiratory diseases [10,11,12,13].

Congenital central hypoventilation syndrome (CCHS) is a rare autosomal dominant genetic disorder (ORPHA:661) that affects respiratory control (with an incidence of about 1/200,000 live births [14,15]. Heterozygous polyalanine repeat expansion (PolyALA) in the paired-like homeobox 2b (*PHOX2B*) gene is present in 90% of CCHS patients [16]. *PHOX2B* is a key gene for the neuronal formation and differentiation, especially in the autonomic nervous system. Indeed, CCHS patients manifest episodes of apnea/hypopnea during sleep and during routine activities of daily living. This pathological condition results from abnormal control of breathing with reduced sensitivity to hypercapnia and hypoxemia [17]. CCHS has no resolving drug therapy [16,18], but lifelong ventilation support during sleep and, in severe cases, also during wakefulness supplants the “forgotten breathing” in CCHS patients [14,15], allowing CCHS patients to live a dignified life.

In 2018, we demonstrated for the first time that erythrocytes and leukocyte subpopulations of CCHS patients had increased levels of ROS [19].

Blood cells reflect the condition of the whole organism and are an outstanding model for evaluating oxidative status, but ROS determination in blood cells requires complex and time-consuming techniques such as cytofluorimetric analysis. Therefore, there is much emphasis on circulating biomarkers of oxidative stress because they can be easily examined in liquid biopsies (e.g., serum and urine), whereas determination of ROS levels requires very rapid sample processing (within 2 h from collection). In light of these considerations, we recently performed an HPLC–MS/MS analysis to detect the three major oxidative biomarkers, i.e., *o*,*o*′-dityrosine, malondialdehyde, and 8-hydroxy-2′-deoxyguanosine, in the CCHS urine. This study showed higher levels of urinary MDA in young adults with CCHS than in control subjects of the same age, but not in minors [20].

According to the assumption that OS contributes to the onset of chronic-degenerative diseases [21,22], the determination of OS in CCHS may indicate the risk of suffering from additional disorders during the course of the CCHS rare genetic disease [23].

Therefore, in this study, circulating antioxidant/oxidant status was evaluated by measuring FRAP, TBARS, AOPP, and AGEs in CCHS patients to evaluate the relationship between OS and CCHS, and to investigate the significance of this balance in this rare genetic disease.

## 2. Materials and Methods

### 2.1. Subject Enrollment and Sample Collection

For this study, four minor healthy controls, five adult healthy controls, and nine CCHS patients (five minors and four adults) with confirmed *PHOX2B* gene mutation were enrolled. Enrolled subjects received detailed explanations of the study, and all subjects signed a written, informed consent form; for minors, a parental written informed consent form was signed. The Institutional Review Board of Meyer Children’s Hospital (Florence, Italy) approved the study protocol (Ethics Committee registration number 99/2017).

All subjects were Italian and of the same ethnicity; they had no differences in height, weight, body mass index (BMI), and lipid profiles. Healthy controls were age-matched subjects without diseases, not taking drugs or alcohol, and nonsmokers. All CCHS patients had no additional phenotypic features of CCHS (i.e., neural crest tumor or Hirschsprung’s disease). Table 1 reports the demographic and clinical features of CCHS patients.

ROS production is influenced by daily activities or individual-dependent factors [24]. CCHS patients were admitted to the hospital the day before sample collection to exclude ROS production due to acute exposure to other factors or daily activities. The respiratory parameters, i.e., apnea–hypopnea index, oxygen desaturation index, and carbon dioxide levels, of all CCHS patients were within normal limits after they underwent full nocturnal polysomnographic examinations and capnography to exclude any possible interference from recent hypoxic episodes.

All blood samples were collected fasting in the morning; for CCHS patients, blood was sampled after the polysomnography analysis. Blood was collected in nonadditive serum preparation tubes (red top). The serum tubes were kept at room temperature for 60 min before centrifugation (1000× *g*, 15 min). Serum samples were stored at −20 °C within 2 h of collection.

### 2.2. Thiobarbituric Acid-Reactive Substances (TBARS) Determination

TBARS are markers of lipid peroxidation, and they were determined as reported in our previous work [25]. Briefly, the serum sample (100 µL) was deproteinized by adding 100 µL of trichloroacetic acid (Merck KGaA, Darmstadt, DA, Germany) (1:1 *v*/*v*), and 160 µL of the resulting supernatant was added to 32 µL of thiobarbituric acid (0.12 M) (Merck KGaA, Darmstadt, DA, Germany) in 0.26 M Tris, before heating at 100 °C for 15 min. A 10 min ice bath and centrifugation (1600× *g*, 4 °C, 10 min) stopped the reaction, and the absorbance of the supernatant was measured at 532 nm (Perkin Elmer Wallac 1420 Victor3 Multilabel Counter).

The molar absorption coefficient (1.56 × 10^−5^ M·cm^−1^) was used to calculate TBARS content. TBARS were expressed as pmol/mg protein.

### 2.3. Advanced Oxidation Protein Product (AOPP) Determination

For AOPP measurement, the serum sample (20 µL) was diluted in 1 mL of PBS, and then mixed with 50 µL of KI (1.16 M) and 100 µL of acetic acid. The absorbance value was immediately read at 340 nm. Chloramine-T (Merck KGaA, Darmstadt, DA, Germany), as the standard for the calibration curve, was used to quantify AOPPs. AOPPs were expressed as nmol of chloramine per mg protein [26].

### 2.4. Advanced Glycated End-Products (AGEs)

According to Cournot and Burillo [27], AGEs were determined by spectrofluorometric detection of fluorescent products. A 96-well black plate was loaded with 100 µL of serum, and fluorescence was measured at 460 nm after excitation at 355 nm. As a result, the results were expressed in arbitrary units (AU)/mg protein.

### 2.5. Ferric Reducing Ability of Plasma (FRAP) Determination

Assays based on Benzie and Strain’s method were used to determine FRAP [28]. As previously described [25], serum samples (10 μL) were added to 90 μL of distilled water. A FRAP solution (300 mM acetate buffer (pH 3.6), 10 mM 2,4,6-tripyridyl-S-triazine (TPTZ) in 40 mM HCl, and 20 mM FeCl_3_·6H_2_O (10:1:1)) was freshly prepared incubated for 30 min at 37 °C. The ferric–tripyridyl–triazine complex is reduced into the ferrous form, developing an intense blue color. The absorbance value was measured at 595 nm using the Wallac 1420 Victor 3 Multilabel Counter (Perkin Elmer, Waltham, MA, United States). FeSO_4_·7H_2_O was used for the standard curve, FRAP was expressed as nmol/mg protein. Merck KGaA (Darmstadt, DA, Germany) supplied all reagents.

### 2.6. Statistical Analysis

Statistical analyses were performed using Graph-Pad Prism 7.00. A *p*-value < 0.05 was considered statistically significant. Normality was verified with the Kolmogorov–Smirnov test. Comparison of continuous variables between the two groups was performed using the Student’s *t*-test or Mann–Whitney test as applicable according to data distribution.

## 3. Results and Discussion

Oxidative Damage and Antioxidant Capacity in the Serum of CCHS Patients

Biomarkers of systemic OS and total antioxidant capacity are of great value in assessing a physiologically altered or increased disease state [29].

As shown in Table 2, the mean age of CCHS patients was similar to that of the controls (15.61 ± 3.3 vs. 17.89 ± 3.62), and the two groups were also matched for sex. AOPP levels were significantly higher in the CCHS serum when compared to age- and sex-matched healthy subjects (12.18 ± 1.81 vs. 2.76 ± 0.72, *p* < 0.001). This finding is consistent with the elevated ROS levels we previously detected in blood cells of CCHS patients [19]. On the contrary, no significant differences were observed when comparing TBARS and AGE levels in the two groups; similarly, the total antioxidant power of CCHS patients was similar to that of the healthy controls.

Since we recently demonstrated increased urinary MDA levels in young adult CCHS compared with age-matched control subjects [20], in this study, we evaluated serum OS biomarkers and antioxidant status according to two age groups: minors (<18 years) and adults (>18 years).

**Figure 1:**
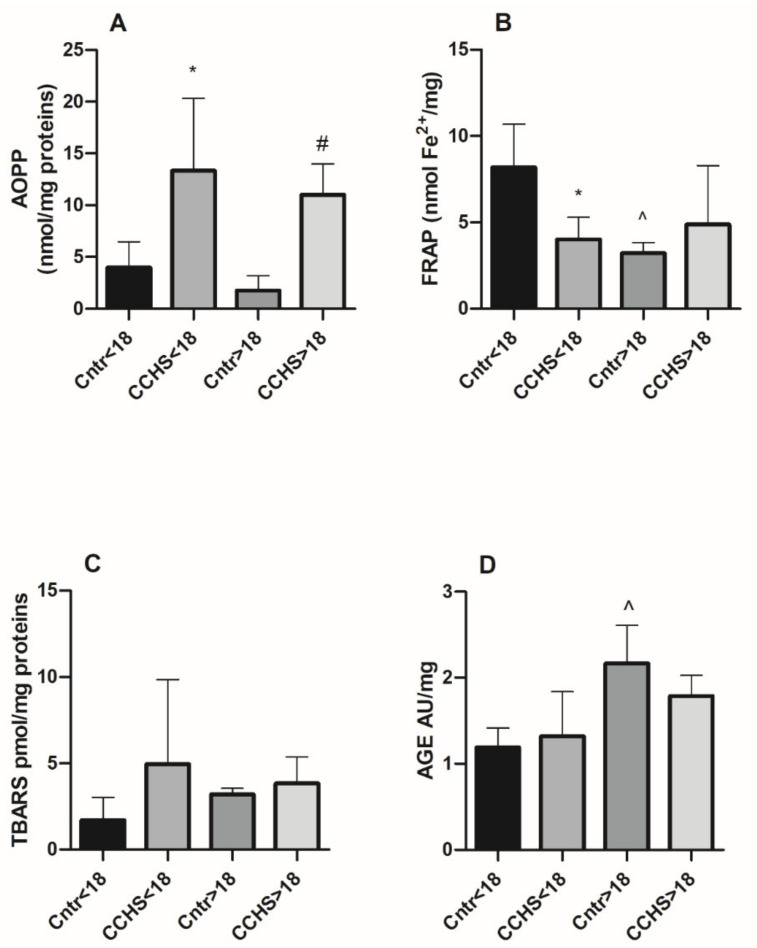
Levels of serum (**A**) AOPP, (**B**) FRAP, (**C**) TBARS, and (**D**) AGEs in healthy controls (Cntr) and CCHS patients aged <18 years and >18 years. Data are expressed as the mean ± SEM. * *p* < 0.05 vs. healthy minors; ^#^ *p* < 0.05 vs. healthy adults; ^ *p* < 0.05 vs. healthy minors.

As depicted in Figure 1A, AOPP levels were significantly higher in both minor CCHS patients (mean age 7.9 ± 1.73 years) compared to age-matched controls (mean age 7 ± 2.16 years) (13.35 ± 3.48 nmol/mg vs. 3.99 ± 1.24 nmol/mg; *p* < 0.05) and adult CCHS patients (mean age 25.25 ± 2.13 years) compared to adult healthy controls (mean age 26.60 ± 1.28 years) (11.01 ± 1.50 nmol/mg vs. 1.78 ± 0.63 nmol/mg; *p* < 0.05).

AOPPs resulting from myeloperoxidase activation and hypochlorous acid production during inflammation are capable of binding to pattern recognition receptors (PRRs) whose downstream effects result in the activation of OS and inflammatory pathways including nicotinamide adenine dinucleotide phosphate (NADPH) oxidase and nuclear factor-κB (NF-κB) activation [30]. Among the PPRs activated by AOPPs, the receptor of advanced glycation end-products (RAGE) is implicated in diverse chronic inflammatory states and has been linked to vascular lesions in diabetes, renal diseases, and atherosclerosis [31,32]; AOPPs have been also found increased in patients with inflammatory conditions such as Crohn’s disease [4], rheumatoid arthritis [33], and obstructive sleep apnea syndrome [34].

In addition, FRAP values were significantly lower in CCHS aged below 18 years compared to healthy minors (4.01 ± 0.57 nmol Fe^2+^/mg vs. 8.17 ± 1.25 nmol Fe^2+^/mg, *p* < 0.05) whereas no difference was observed when comparing FRAP levels from adult CCHS to adult controls (Figure 1B). Comparing FRAP values between healthy subjects, adults showed significantly lower levels compared to minors (3.22 ± 0.26 nmol Fe^2+^/mg vs. 8.17 ± 1.25 nmol Fe^2+^/mg, *p* < 0.05). Reports on FRAP values in healthy children and young adults are lacking, and data on antioxidants plasma levels are contradictory. The concentration of total thiols in the plasma of females, but not of males, progressively decreased in the group aged 20–39 compared to groups aged 6–13 and 14–19 [35]; on the contrary, a slight increase in total antioxidant capacity, measured by TAC assay, was observed in the plasma of healthy children (aged 2–14) compared to adults (aged 25–45) [36]. FRAP levels are indicative of antioxidants such as ascorbic acid, α-tocopherol, uric acid, and bilirubin, while thiols (including GSH and albumin) react slowly in this assay [9]. The relative contribution of each single antioxidant that may react in the FRAP assay deserves to be further evaluated to understand which specific antioxidant may account for the decreased FRAP levels observed in minor CCHS patients. This further analysis could also shed light on the physiological processes responsible for the reduced FRAP values in young healthy adults compared to healthy minors.

However, our data suggest that, in CCHS minors, nonenzymatic antioxidant sources were impaired, which could have contributed to the increased AOPP levels detected in these patients. We can hypothesize that while, in minors, the depletion of antioxidants could be a major contributor to AOPP formation, in adults, this seems more linked to a direct activation of inflammatory processes.

In our previous study, increased urinary MDA levels, detected by means of an HPLC–MS/MS analysis, were demonstrated in young CCHS adults compared with controls [20]. In this study, it was found that serum TBARS were not affected by the clinical status in minors or in adults (Figure 1C).

This could be explained by the different biological fluids analyzed (serum vs. urine), but most probably by the detection method; in fact, spectrophotometric TBARS determination is nonspecific for MDA since the thiobarbituric acid test measures reactive substances other than MDA, such as 2-alkenals and 2,4-alkadienals [37]. It was also demonstrated that the lack of specificity of the spectrophotometric TBARS determination limited the detection of differences in the level of lipid peroxidation in biological samples observed by HPLC detection [38].

Furthermore, this study showed that AGEs levels were not altered between patients with CCHS and healthy controls but were increased in healthy adults compared with healthy minors (Figure 1D). This event is in line with the knowledge of AGEs increase with advancing age, including the transition from pediatric to adult age, contributing to the aging process [39]. Another interesting aspect, which deserves further evaluation, is the lack of significant differences in all the measured parameters between minor and adult CCHS patients. This result suggests that OS, particularly the AOPP/RAGE axis, which operates at the crossroad between OS and inflammation, might be intrinsically linked to the CCHS pathogenesis and the role of *PHOX2B* mutations in this disease.

Overall, our results suggest increased OS in CCHS patients and that AOPPs are the most relevant serum biomarker for monitoring OS in these patients. It is noteworthy that AOPPs can cause cellular dysfunction, cell death, and tissue damage in different organs, thus representing promising biomarkers of the redox imbalance associated with CCHS and its complications.

In this study, the enrolled patients did not have hypoventilation during wakefulness; the apnea index and dyspnea index, oxygen desaturation index, and carbon dioxide levels in these patients were in the normal range during night ventilation. Therefore, we can rule out an association between the results obtained in this study and the possible ineffectiveness of artificial ventilation and sleep quality. However, to date, very little is known about the downstream cellular and molecular consequences of *PHOX2B* mutations, although some studies have suggested that transcriptional dysregulation and protein misfolding may underlie pathogenesis [18,40]

## 4. Conclusions

The biochemical mechanisms underlying rare diseases are generally poorly understood. In most cases, the chronic pathophysiological conditions associated with the disease, in addition to the patient’s clinical manifestations, are generally asymptomatic. However, they could be responsible for the onset of other chronic collateral disorders causing patients’ clinical worsening over the lifetime.

Discoveries about molecules or signaling pathways associated with these latent pathophysiological processes could provide fundamental knowledge to intervene early against worsening clinical status.

This communication adds new insights about the systemic oxidative state in CCHS patients and demonstrates for the first time that AOPPs are one of the key serum biomarkers of oxidation in CCHS.

Because AOPPs are known to be stimulating factors of inflammatory responses, this study suggests a possible involvement of the inflammatory process in CCHS. In light of these considerations, further evaluation on the inflammatory state in these patients could contribute to the understanding of the pathophysiology of this rare genetic disease.

Overall, the results obtained so far on CCHS generate hypotheses for future clinical trials aimed at improving the CCHS outcomes by hindering increased oxidative stress through the promotion of a healthy lifestyle, adequate diet, and the possible therapeutic use of antioxidant agents or pharmacological strategies targeting the AOPP/RAGE axis and activation of its downstream inflammatory pathways.

## Figures and Tables

**Table 1:** Demographic and clinical characteristics of enrolled CCHS patients.

ID	Age	Sex	*PHOX2B* Gene Mutation	Type of Ventilation
3	30	F	PolyALA 20/25	Pacemaker/noninvasive ventilation
8	26	M	PolyALA 20/26	Pacemaker/noninvasive ventilation
7	20	F	PolyALA 20/33	Pacemaker/noninvasive ventilation
2	27	F	PolyALA 20/25	Noninvasive ventilation
5	8	M	PolyALA 20/25	Noninvasive ventilation
1	11	F	PolyALA 20/26	Tracheostomy
6	11	M	PolyALA 20/26	Tracheostomy
4	1	F	Frameshift	Tracheostomy
9	11	M	PolyALA 20/25	Noninvasive ventilation

**Table 2:** Demographic features, and serum biomarkers in CCHS patients and healthy controls.

Demographic Characteristics and Oxidative Markers	CCHS Patients	Healthy Controls
*n*	9	9
Sex (males, %)	44%	33%
Age (years)	15.61 ± 3.3	17.89 ± 3.62
TBARS (pmol/mg)	4.46 ± 1.21	2.52 ± 0.38
AOPP (nmol/mg)	12.18 ± 1.81 ***	2.76 ± 0.72
FRAP (nmol Fe^2+^/mg)	4.40 ± 0.77	5.42 ± 1.01
AGEs (AU/mg)	1.63 ± 0.12	1.84 ± 0.22

Data are reported as the mean ± SE. *** *p* < 0.001 vs. healthy controls according to Student’s *t*-test. TBARS: thiobarbituric acid-reactive substances; AOPPs: advanced oxidation protein products; FRAP: ferric reducing ability of plasma; AGEs: advanced glycation end-products; AU: arbitrary units.

## Data Availability

Data is contained within the article.
